# Hereditary Transthyretin Amyloidosis: How to Differentiate Carriers and Patients Using Speckle-Tracking Echocardiography

**DOI:** 10.3390/diagnostics13243634

**Published:** 2023-12-09

**Authors:** Daniela Di Lisi, Filippo Brighina, Girolamo Manno, Francesco Comparato, Vincenzo Di Stefano, Francesca Macaione, Giuseppe Damerino, Leandro Di Caccamo, Noemi Cannizzo, Antonella Ortello, Alfredo R. Galassi, Giuseppina Novo

**Affiliations:** 1 Division of Cardiology, University Hospital Paolo Giaccone, 90127 Palermo, Italygdamerino@gmail.com (G.D.);; 2 Department of Health Promotion, Mother and Child Care, Internal Medicine and Medical Specialties (PROMISE) “G. D’Alessandro”, University of Palermo, 90127 Palermo, Italy; 3 Section of Neurology, Department of Biomedicine, Neuroscience and Advanced Diagnostic (BIND), University of Palermo, 90127 Palermo, Italy

**Keywords:** cardiac amyloidosis, apical sparing, speckle-tracking echocardiography, transthyretin

## Abstract

Background: Hereditary transthyretin amyloidosis is a rare disease caused by transthyretin (TTR) gene mutations. The aim of our study was to identify early signs of cardiac involvement in patients with a TTR gene mutation in order to differentiate carriers from patients with neurological or cardiac disease. Methods: A case–control study was carried out on 31 subjects with the TTR mutation. Patients were divided into three groups: 23% with cardiac amyloidosis and polyneuropathy (group A), 42% with only polyneuropathy (group B) and 35% carriers (group C). Speckle-tracking echocardiography (left-ventricular global longitudinal strain—GLS, atrial stiffness) was performed in all patients. The apical/basal longitudinal strain ratio (SAB) and relative apical sparing (RAS) were assessed in all subjects. Results: Analyzing groups C and B, we only found a significant difference in the SAB (*p*-value 0.001) and RAS (*p*-value 0.039). These parameters were significantly more impaired in group A compared to group B (SAB *p*-value 0.008; RAS *p*-value 0.002). Also, atrial stiffness was significantly impaired in groups A and B compared to group C. Conclusions: Our study suggests the diagnostic role of the SAB and RAS in cardiac amyloidosis. The SAB and RAS showed a gradual increase from carriers to patients with neurological and cardiac diseases. Thus, these parameters, in addition to atrial stiffness, could be used to monitor carriers. More extensive data are needed.

## 1. Introduction

Cardiac amyloidosis, a rare disease until a few years ago, is becoming increasingly recognized and diagnosed. In the past, the diagnosis of amyloidosis was made after many specialist visits because diagnostic strategies were not known [1].

At present, several “red flags” are known to increase the diagnostic suspicion of amyloidosis [2], and amyloidosis should be looked for in patients with these “red flags” because a timely diagnosis and treatment improve prognosis [3,4].

There are various types of cardiac amyloidosis. The most common are: light-chain (AL) amyloidosis associated with the presence of multiple myeloma [5] and cardiac transthyretin amyloidosis (ATTR), either wild-type (non-mutated) transthyretin (ATTRwt) or variant transthyretin (ATTRv) [6].

Hereditary transthyretin amyloidosis (ATTRv amyloidosis) is a rare, autosomal-dominant disease. It is caused by pathogenic variants in the transthyretin (TTR) gene, and it can progressively involve several organs [7,8]. In particular, cardiac amyloidosis, which occurs when misfolded amyloid fibrils are deposited in the myocardium, is an increasingly recognized cause of heart failure with a preserved ejection fraction (HFpEF) [9]. Cardiac amyloidosis may be associated with other cardiovascular complications such as aortic stenosis, ventricular and supraventricular arrhythmias, bradyarrhythmias, and increased thromboembolic risk. The treatment of these cardiovascular complications is not always easy [10,11].

Several TTR mutations exist, and there is a notable genotype–phenotype variability [12]. Some mutations are often associated with predominant polyneuropathy, such as V30M. Other mutations, such as V122I, are often associated with predominant cardiomyopathy; other mutations are associated with a mixed phenotype [13].

There is also considerable variability in the penetrance, and some carriers may never develop cardiac or neurological disease or may develop them late. Thus, it is very important to follow patients with a TTR mutation without overt cardiac amyloidosis but with a neurological phenotype or carriers over time in order to detect early signs of possible cardiac involvement.

Echocardiography allows for suspicion of cardiac amyloidosis if there is an unexplained increase in the wall thickness and other cardiac and extracardiac signs defined as “red flags” (including extracardiac and cardiac signs, laboratory tests, electrocardiogram—ECG, echocardiography and cardiac magnetic resonance—CMR) [14].

Recently a multiparametric echocardiographic score (increased wall thickness score—IWT score) has been proposed to identify patients with a higher probability of cardiac amyloidosis compared to other types of hypertrophic cardiomyopathy [15].

The IWT score includes the relative wall thickness (RWT), E’/e’ ratio, tricuspid annular plane systolic excursion (TAPSE), left-ventricular global longitudinal strain (GLS) and apical/basal strain ratio (SAB) [15]. In particular, the presence of multiple altered echocardiographic parameters (for example, RWT > 0.6; E/e’ > 11, TAPSE ≤ 19 mm, GLS ≥ −13% and SAB > 2.9) with an IWT score ≥ 8 makes the diagnosis of cardiac amyloidosis probable, an IWT score between 2 and 7 requires further investigation and an IWT score < 2 makes the diagnosis of cardiac amyloidosis unlikely [15].

In fact, in patients with cardiac amyloidosis, right-ventricular function is also impaired with hypertrophy of the free-wall right ventricle; the thickness of the valves increases, and aortic stenosis can be associated with cardiac amyloidosis [10].

Among the echocardiographic parameters, a relative apical sparing (RAS, calculated as the average apical longitudinal strain/average basal + average mid-longitudinal strain) = 1 shows a high sensitivity and specificity in differentiating patients with cardiac amyloidosis from patients with left-ventricular hypertrophy [16].

Thus, speckle-tracking echocardiography (STE) plays a very important role in differentiating cardiac amyloidosis from other types of left-ventricular hypertrophy [17,18]. In fact, in patients with cardiac amyloidosis, the longitudinal strain is more reduced in the basal segments compared to the apical ones (apical sparing). Various mechanisms have been hypothesized to explain this apical sparing.

For example, less amyloid deposition at the apex rather than the base resulting in the relative sparing of apical longitudinal fibril contraction, the greater diversity of myocyte and matric orientation at the apex compared with the base that can potentially have a role in the preservation of the apical longitudinal strain or the greater tendency toward apoptosis and remodeling in the basal segments related to turbulent flow in the LV outflow tract and a higher parietal stress [19].

Furthermore, other studies have also demonstrated an elevated LVEF/GLS ratio in patients with cardiac amyloidosis [20].

Other studies have shown the role of the left-ventricular (LV) mass-to-GLS ratio (mass-to-strain ratio (MSR)) in determining cardiac amyloidosis subtypes. MSR was also shown to be the best discriminator between ATTR and AL cardiomyopathies compared with RAS and LVEF/GLS [21].

The suspicion of cardiac amyloidosis can be confirmed by cardiac or extracardiac biopsy + echocardiography/CMR criteria. Only for ATTR amyloidosis can the diagnosis be carried out non-invasively if there is grade 2 or 3 cardiac uptake on the diphosphonate bone scintigraphy, negative hematological tests and echocardiographic/CMR criteria suggestive for cardiac amyloidosis [14,22].

Recent progress in the pharmacological treatment of cardiac amyloidosis has inaugurated a new era in the management of the disease and highlighted the importance of making an early diagnosis of cardiac involvement [23,24].

In fact, beginning treatment at an early stage allows for stopping the evolution of the disease before irreversible organ damage occurs and can improve prognosis.

Considering that the diagnosis of cardiac amyloidosis can be performed in various stages of the disease and considering that carrier patients can develop cardiac amyloidosis, the aim of our study was to analyze the main echocardiographic and STE differences between carriers, neuropathic patients and patients with cardiac amyloidosis in order to identify the parameters more associated with the presence and/or development of cardiac amyloidosis.

## 2. Material and Methods

A retrospective cross-sectional study was carried out by enrolling patients with the TTR gene mutation with or without neurological or cardiac disease. The enrolled patients were followed at the Cardiology Unit and Neurology Unit of the University Hospital Paolo Giaccone in Palermo (Italy) from 2020 to 2022. All patients signed informed consent. The study was conducted in accordance with the Declaration of Helsinki and approved by the Institutional Review Board (or Ethics Committee) of Policlinico P. Giaccone Palermo (V. n.7/2020).

The inclusion criteria were:

- TTR gene mutation able to cause ATTRv amyloidosis;
- Age between 18 and 80 years old;
- Any stage of cardiac amyloidosis;
- Any stage of polyneuropathy.

The exclusion criteria were:

- Patients with ATTRwt cardiac amyloidosis;
- Patients with AL cardiac amyloidosis and multiple myeloma;
- Bad acoustic window to perform STE.

On the basis of the phenotype, the patients were divided into 3 groups:

- Group A: patients with both cardiac amyloidosis and neurological involvement.
- Group B: patients with only polyneuropathy.
- Group C: carriers.

The cardiological evaluation included a clinical examination, an electrocardiogram with QTc interval measurement and a color Doppler echocardiogram with STE analysis. Assessment of cardiovascular risk factors (arterial hypertension, dyslipidemia, diabetes mellitus, smoking, obesity) and cardiovascular comorbidity (including ischemic heart disease, peripheral arteriopathies, transient ischemic attack-TIA and stroke) was performed. Major anthropometric/functional variables and drug history were recorded (weight, height, BSA, BMI, NYHA class). The echocardiographic examination was performed according to the recommendations of the American Society of Echocardiography/European Association of Cardiovascular Imaging for chamber quantification and diastolic function evaluation [25,26].

Accordingly, the left-ventricular ejection fraction (LVEF) and diastolic function were assessed, atrial volumes were measured and the right-ventricular function was assessed by measuring the tricuspid annular plane systolic excursion (TAPSE). Speckle-tracking echocardiography analysis was performed. The left-ventricular global longitudinal strain (GLS), peak atrial longitudinal strain (PALS) and atrial stiffness were measured after acquiring apical two-chamber, three-chamber and four-chamber views with a frame rate between 60–90 frames per second. Analysis was performed offline using a semi-automated 2D-strain software package (EchoPacV.202, GE Healthcare, Horten, Norway). The left-ventricular endocardial border was manually traced in the apical views, delineating a region of interest (ROI) of six segments for each view. Then, necessary manual adjustments of the ROI were performed, and the longitudinal strain curves for each segment were generated by the software. The GLS was calculated as the average of the four-chamber, two-chamber, and three-chamber longitudinal strain curves.

In addition, we assessed the apex-to-base ratio of the longitudinal strain (SAB: average apical longitudinal strain/average basal longitudinal strain) and relative apical sparing (RAS: average apical longitudinal strain/sum of the average basal and mid longitudinal strain) [16,27,28,29].

The PALS was measured after manual tracing of the left atrium endocardial border using a dedicated acoustic tracking software for the left atrium (EchoPacV.202, GE Healthcare, Horten, Norway, version for LA strain analysis: 2.02—release 34.0).

The PALS was defined as the first peak positive deflection and is representative of the LA reservoir function. The PALS was calculated as the mean value of 2 apical views (4 and 2 chambers). We used R-R gating as the zero-reference point.

Atrial stiffness was defined as: early diastolic transmitral inflow velocity/mitral annulus early diastolic velocity and PALS ((E/e′)/PALS).

Diagnosis of cardiac ATTR amyloidosis was performed according to the position statement of the European Society of Cardiology [14].

### Statistical Analysis

The data were collected in an Excel 2021 database, and their statistical analysis was carried out through the same software, Graphpad Prism 8.2.1 and Medcalc Statistical Software version 19.1.7.

The categorical variables were expressed as the absolute frequency (n°) and percentage (%), while the continuous variables were expressed as the mean and standard deviation (SD). Differences between continuous variables were derived using Student’s t-test. A *p*-value ≤ 0.05 was considered statistically significant.

## 3. Results

The study included 31 patients (35.5% women and 64.5% men; median age 61.90 ± 10.67 years old), including 7 patients (23%) with both cardiac amyloidosis and amyloid polyneuropathy, 13 patients (45%) with amyloid polyneuropathy and 11 (35%) carriers.

**Figure 1:**
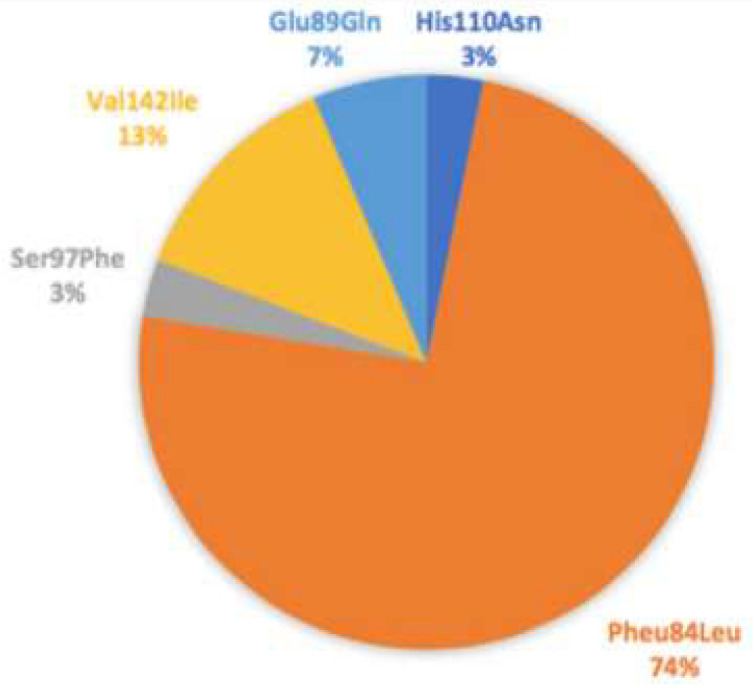
ATTR gene mutation.

The Phe64Leu ATTR gene mutation was the most represented mutation in the population (71.2% of patients). A total of 3.2% of patients had the His110Asn gene mutation, 3.2% of patients had the Ser97Phe mutation, 12.8% of patients had the Val122Lle mutation and 6.4% of patients had the Glu89Gln gene mutation (Figure 1).

Demographic, clinical and laboratory characteristics of the three groups of patients are reported in Table 1.

There were no significant differences regarding cardiovascular risk factors between the various groups (Table 1).

In the group with cardiac amyloidosis (group A), atrial fibrillation was present in 28% of the patients. In the group with amyloid neuropathy, atrial fibrillation was present in 7% of the patients, while none of the carriers presented it.

Analyzing group B (patients with polyneuropathy) and group C (carriers), we found significantly increased values of NT-proBNP in group B compared to group C (14.03 ± 3.13 vs. 285 ± 379; *p*-value 0.02).

Also, between groups A and B, we found significant differences in the values of NT-proBNP (1168.8 ± 513.7 vs. 285 ± 379; *p* value 0.0003).

Increased values of troponin Ths were present in group A compared to the other groups; between groups A and B, we found significant differences (26.7 ± 5 vs. 4.47 ± 5.40; *p* value < 0.0001).

The derived EGFR values (CKD-EPI) were statistically different between group C and group B (107.87 ± 39.30 vs. 67.53 ± 21.27; *p*-value = 0.004) and between group B and group A (67.53 ± 21.27 vs. 88.33 ± 16.26; *p*-value = 0.03).

There are no significant differences between group C and group B concerning the NYHA class, while all the patients in group A were at least in the NYHA II class (Table 1).

### 3.1. Echocardiographic Characteristics

Analyzing the echocardiographic data (Table 2) and comparing the groups, we found significant differences between the patients with cardiac amyloidosis and the other groups, but we also found significant differences between the carriers and patients with the TTR gene mutation with only neurological involvement. In particular, we made two comparisons:

- The population with cardiac amyloidosis vs. that with amyloid polyneuropathy (group A vs. group B)
- The population with amyloid polyneuropathy vs. carriers (groups B vs. group C).

### 3.2. Population with Amyloid Polyneuropathy vs. Carriers

The carriers and patients with only polyneuropathy had normal LVEF values, diastolic function and wall thicknesses (Table 2). Comparing groups B and C, we did not find significant differences in the LVEF and diastolic function values. We found lower values of the GLS in the patients with polyneuropathy compared to the carriers, even if the difference was not statistically significant (−17.6 ± 4 vs. −19.7 ± 1.6; *p*-value = 0.11). We found significantly increased values of the SAB (1.58 ± 0.25 vs. 1.33 ± 0.20; *p*-value = 0.008) and RAS (0.72 ± 0.08 vs. 0.62 ± 0.07; *p*-value = 0.0039) in group B compared to group C.

**Figure 2:**
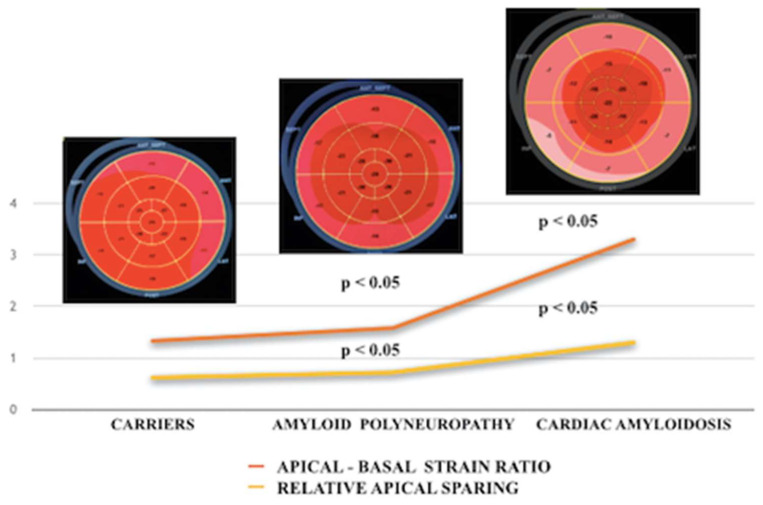
Progression of SAB and RAS from carriers to amyloid neuropathy and cardiac amyloidosis. SAB = apical-basal strain ratio; RAS = relative apical sparing.

Thus, we observed small differences between the carriers and patients with polyneuropathy. In particular, we observed a gradual reduction in the GLS (not significant) and a significant increase in the SAB and RAS between the carriers and patients with polyneuropathy (Figure 2).

We found significant differences between group B and group C concerning first-degree (*p*-value = 0.05) and second-degree diastolic dysfunction (*p*-value = 0.05).

Between groups B and C, a significant reduction in the septal E′ (10.8 ± 3.6 vs. 8 ± 1.8; *p*-value = 0.02), lateral E′ (15 ± 3.8 vs. 9.7 ± 2.5; *p*-value = 0.0005) and lateral S′ (13.4 ± 3.6 vs. 10.5 ± 3.4; *p*-value = 0.05) values and an increase in the E/e′ ratio (7.6 ± 3 vs. 5.3 ± 1; *p*-value = 0.02) were detected.

In addition, we did not find any significant difference in the PALS between groups B and C (23.63 ± 11.20 vs. 23.64 ± 10.06; *p*-value = 0.99), but we found a significant difference in atrial stiffness between these groups (0.58 ± 0.2 vs. 0.299 ± 0.21; *p*-value = 0.002).

We did not find any significant differences in the right-ventricular function and dimensions between these two groups (Table 2).

### 3.3. Population with Cardiac Amyloidosis vs. Amyloid Polyneuropathy

Group A showed the typical echocardiographic features of cardiac amyloidosis, as expected. We found reduced values of LVEF (51.9 ± 6.7 vs. 59.8 ± 5; *p*-value = 0.007), increased left-ventricle end diastolic pressures (E/e′ 15 ± 3.8 vs. 9.7 ± 2.5; *p*-value = 0.0001), reduced right-ventricle indices of longitudinal function (TAPSE 16.3 ± 5.4 vs. 21.9 ± 3.4; *p*-value = 0.01), increased wall thicknesses (16.4 ± 4.1 vs. 10.5 ± 2; *p*-value = 0.0002), increased left atrial volume (86.1 ± 29.4 vs. 56.9 ± 14; *p*-value = 0.007) and left atrial volume indexed (50 ± 8 vs. 33.4 ± 5, *p*-value < 0,0001), reduced GLS values (12.5 ± 3.9 vs. 17.6 ± 4; *p*-value = 0.01) and significantly increased SAB (3.3 ± 2.1 vs. 1.58 ± 0.25; *p*-value = 0.008) and RAS (1.3 ± 0.6 vs. 0.72 ± 0.08; *p*-value = 0.0025) values in group A compared to group B (Table 2). We did not find a significant difference between the prevalence of types I and II diastolic dysfunctions between group A and group B, but we found type III diastolic dysfunction only in group A.

**Figure 3:**
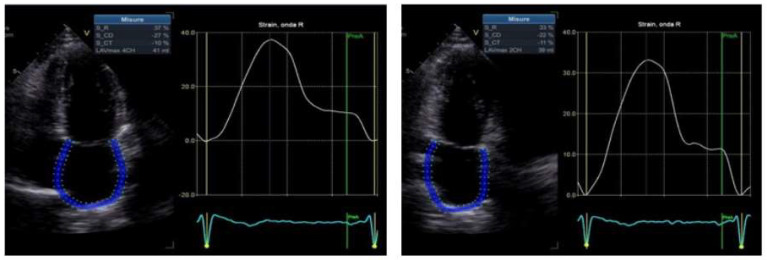
Atrial strain.

In addition, the patients with cardiac amyloidosis had significantly reduced PALS values compared to the patients with only polyneuropathy (9.8 ± 3 vs. 23.63 ± 11.2; *p*-value = 0.005) and significantly increased atrial stiffness values (1.6 ± 0.30 vs. 0.58 ± 0.2; *p*-value < 0.0001), see Figure 3 and Table 2.

Thus, the patients with cardiac amyloidosis had significant changes in their systolic and diastolic function and STE parameters compared to groups B and C.

In particular, we found gradual and progressive changes in the STE parameters in the patients with various stages of ATTR amyloidosis (from carriers to neuropathic patients to patients with neuropathy plus cardiac amyloidosis). Above all, the SAB and RAS values increased significantly in the various stages of advanced pathology, LASI increased significantly and GLS decreased, see Figure 2.

## 4. Discussion

Our study confirms the diagnostic role of the SAB and RAS in cardiac amyloidosis, which is in agreement with the literature data [16,19]. The patients with cardiac amyloidosis had significant echocardiographic impairments compared to the patients with only polyneuropathy and the carriers.

The GLS, SAB, RAS, PALS and atrial stiffness presented different degrees of alteration in the various stages of amyloidosis (from carriers to neuropathic patients to patients with neuropathy plus cardiac amyloidosis).

In particular, in the patients with cardiac amyloidosis, the SAB and RAS values were significantly increased and the GLS was significantly reduced compared to the patients with only polyneuropathy and the carriers. In addition, the PALS and atrial stiffness were also significantly altered compared to the other patients.

Our study showed that the patients with the TTR gene mutation without cardiac involvement at transthoracic echocardiogram on the basis of the current known criteria (low IWT score, absence of increased left-ventricular wall thickness, normal values of tissue velocities and normal GLS, normal diastolic function) also had decreased GLS value; in particular, we observed a gradual increase in the SAB and RAS from carriers to patients with neurological involvement and to patients with cardiac amyloidosis.

We also observed gradual changes in the PALS and atrial stiffness, progressing from carriers to polyneuropathics and patients with cardiac amyloidosis.

The monitoring of these STE parameters could probably be useful to highlight the progression and/or development of the disease in patients with ATTR amyloidosis, especially in patients with only the gene mutation with or without polyneuropathy.

In addition, we also found a gradient in the values of NT-proBNP from carriers to patients with only polyneuropathy to patients with cardiac amyloidosis. It is known that in patients with amyloidosis, the longitudinal strain is earlier and is greatly reduced, especially at the level of the basal segments, while the apical segments tend to be spared [16,19].

Furthermore, Phelan et al. demonstrated that a relative apical sparing of the longitudinal strain has a high sensitivity and specificity in differentiating patients with cardiac amyloidosis from those with other types of hypertrophy [16].

Therefore, it is consolidated by the literature that the basal segments of the left ventricle are those affected first by the amyloid deposition and by the reverse remodeling [19].

Studies have shown that atrial function is impaired in patients with cardiac amyloidosis [30].

For example, Aimo et al. showed that lower values of the PALS and left-atrial peak atrial contraction strain (PACS) were associated with a high likelihood of cardiac amyloidosis and ATTR-cardiac amyloidosis [29].

Other studies have shown the role of atrial strain in predicting atrial fibrillation in patients with cardiac amyloidosis [31].

Also, the assessment of atrial function in patients with cardiac amyloidosis is very important in evaluating the need for anticoagulant therapy in patients with sinus rhythm and electromechanical dissociation [32,33]. It is known that patients with cardiac amyloidosis have a high thromboembolic risk and that anticoagulant therapy is needed in patients with atrial fibrillation regardless of the CHA2DS2 VASC score [34].

The presence of a progressive alteration in the atrial stiffness indices in the patients with polyneuropathy compared to the carriers could demonstrate how important it is in patients with polyneuropathy to evaluate atrial strain and not only the GLS to highlight early cardiac involvement.

In carriers, it is desirable to start follow-up 10 years before the expected age of disease onset. Various follow-up schemes for carriers have been proposed, including performing a Color Doppler echocardiogram, CMR, ECG-Holter and NT-proBNP [35]. Early diagnosis of cardiac amyloidosis is important because the use of new drugs is improving the survival of patients with ATTR amyloidosis [36].

Cardiological follow-up in patients with amyloid polyneuropathy could be considered, on the basis of the Apollo Trial, every 9 months [37].

Our data support the use of speckle-tracking echocardiography not only in the diagnosis of cardiac amyloidosis but also in the monitoring of carriers and patients with only polyneuropathy.

Carriers may never develop cardiac disease on the basis of the genetic mutation, but certainly a multiparametric echocardiographic evaluation, including the GLS, RAS, SAB and atrial stiffness, could be of help in these patients to detect possible early signs of any future development of cardiac disease.

## 5. Conclusions

Our study suggests the usefulness of speckle-tracking echocardiography not only in patients with cardiac amyloidosis but also in carriers and patients with only amyloid polyneuropathy in detecting possible early signs of cardiac involvement.

A multiparametric echocardiographic evaluation, including the RAS, SAB and atrial stiffness, could be used in these patients during follow-up.

Our study could have important practical implications because the early diagnosis of cardiac involvement in carriers and patients with polyneuropathy would allow for the implementation of effective therapeutic strategies that are revolutionizing the treatment and prognosis of cardiac amyloidosis. Unfortunately, our results are preliminary data, and they cannot be generalized. More extensive data are needed.

### Study Limitations

A major limitation of the present study is the small number of patients with a very small number of patients included in each group, but the study was a single-center study and the disease analyzed is a rare disease.

Therefore, our results should be interpreted carefully and cannot be generalized to all types of mutations because some carriers and neuropaths may never develop cardiac disease. Thus, further studies are necessary to identify how fast the disease progresses from carriers to cardiac amyloidosis. An other limitation is the absence of clinical and echo follow-up.

## Figures and Tables

**Table 1:** General population. BSA = body surface area; EGFR = estimated glomerular filtration rate.

	Group C Carriers (11 Patients)	Group B Amyloid Polyneuropathy (13 Patients)	Group A Cardiac Amyloidosi s (7 Patients)	Unaffected Carrier vs. Amyloid Polyneuropathy	Amyloid Polyneuropathy vs. Cardiac Amyloidosis
Age	54 ± 11	65 ± 7.8	66 ± 8.4	*p* = 0.009	*p* = 0.79
Male	9 (91%)	4 (30%)	7 (100%)	*p* = 0.003	*p* = 0.003
BSA (mq)	1.9 ± 0.2	1.7 ± 0.3	1.8 ± 0.2	*p* = 0.07	*p* = 0.44
Arterial hypertension	3 (27%)	6 (46%)	3 (43%)	*p* = 0.34	*p* = 0.9
Diabetes	1 (9%)	1 (7%)	0	*p* = 0.85	*p* = 0.48
Smoking	2 (19%)	2 (15%)	1 (14%)	*p* = 0.79	*p* = 0.95
Dyslipidae mia	4 (36%)	6 (46%)	2 (28%)	*p* = 0.62	*p* = 0.44
NYHA (I/II)	0	2 (15%)	6 (86%)	*p* = 0.18	*p* = 0.0026
NYHA (III/IV)	0	0	1 (14%)	/	*p* = 0.17
NT-PROBNP ng/L	14.03 ± 3.13	285 ± 379	1168.8 ± 513.7	*p* = 0.02	*p* = 0.0003
TROPONIN-HS ng/L	2.10 ± 3.25	4.47 ± 5.40	26.7 ± 5	*p* = 0.21	*p* < 0.0001
EGFR (CKD-EPI) mL/min	107.87 ± 3930	67.53 ± 21.27	88.33 ± 16.26	*p* = 0.004	*p* = 0.03

**Table 2:** Echocardiographic parameters. IVS = interventricular septum; LVEF = left-ventricular ejection fraction; PW = posterior wall; LA vol = left atrial volume; TAPSE = tricuspid annular plane systolic excursion; LV GLS = left-ventricular global longitudinal strain; PALS = peak atrial longitudinal strain.

Echocardiographic Parameters	Group C Carriers (11 Patients)	Group B Amyloid Polyneuropathy (13 Patients)	Group A Cardiac Amyloidosi s (7 Patients)	Unaffected Carrier vs. Amyloid Polyneuropathy	Amyloid Polyneuropathy vs. Cardiac Amyloidosis
IVS (mm)	10.3 ± 1.6	10.5 ± 2.1	16.9 ± 4.1	*p* = 0.79	*p* = 0.0002
PW (mm)	8.6 ± 1.3	9.1 ± 1.7	14.4 ± 3	*p* = 0.43	*p* = 0.0001
LVEF 2D (%)	61.6 ± 3	59.8 ± 5	51.9 ± 6.7	*p* = 0.30	*p* = 0.007
LA VOL (mL)	54.2 ± 15.6	56.9 ± 14	86.1 ± 29.4	*p* = 0.65	*p* = 0.007
LA vol indexed (mL/mq)	30 ± 4	33.4 ± 5	50 ± 8	*p* = 0.08	*p* < 0.0001
Septal E′ (cm/s)	10.8 ± 3.6	8 ± 1.8	4.7 ± 1.9	*p* = 0.02	*p* = 0.001
Septal S′ (cm/s)	10.3 ± 2.3	9.5 ± 3.2	5.3 ± 1.6	*p* = 0.49	*p* = 0.004
Lateral E′ (cm/s)	15 ± 3.8	9.7 ± 2.5	5.3 ± 1.3	*p* = 0.0005	*p* = 0.0004
Lateral S′ (cm/s)	13.4 ± 3.6	10.5 ± 3.4	6.6 ± 1.8	*p* = 0.05	*p* = 0.01
E/e′ average	5.3 ± 1	7.6 ± 3	16.4 ± 4.8	*p* = 0.02	*p* = 0.0001
TAPSE (mm)	23.7 ± 4	21.9 ± 3.4	16.3 ± 5.4	*p* = 0.24	*p* = 0.01
LV GLS 2D (%)	19.7 ± 1.6	17.6 ± 4	12.5 ± 3.9	*p* = 0.11	*p* = 0.01
sPAP	21.3 ± 4.8	22 ± 8.8	36.2 ± 18.5	*p* = 0.81	*p* = 0.03
Apical–basal strain ratio	1.33 ± 0.20	1.58 ± 0.25	3.3 ± 2.1	*p* = 0.001	*p* = 0.008
Relative apical sparing	0.62 ± 0.07	0.72 ± 0.08	1.3 ± 0.6	*p* = 0.0039	*p* = 0.002
PALS	23.64 ± 10.06	23.63 ± 11.2	9.8 ± 3	*p* = 0.99	*p* = 0.005
Atrial stiffness	0.29 ± 0.21	0.58 ± 0.2	1.6 ± 0.30 0.58 ± 0.2	*p* = 0.002	*p*< 0.0001

## Data Availability

The data presented in this study are available on request from the corresponding author.
